# Low-Intensity Blue Light Supplemented during Photoperiod in Controlled Environment Induces Flowering and Antioxidant Production in Kalanchoe

**DOI:** 10.3390/antiox11050811

**Published:** 2022-04-21

**Authors:** Jingli Yang, Jinnan Song, Byoung Ryong Jeong

**Affiliations:** 1 Department of Horticulture, Division of Applied Life Science (BK21 Four), Graduate School of Gyeongsang National University, Jinju 52828, Korea; yangmiaomiaode@gmail.com (J.Y.); jinnansong93@gmail.com (J.S.); 2 Institute of Agriculture and Life Science, Gyeongsang National University, Jinju 52828, Korea; 3 Research Institute of Life Science, Gyeongsang National University, Jinju 52828, Korea

**Keywords:** antioxidant, supplementation of low-intensity blue light, flower bud formation, photoperiod, photosynthesis

## Abstract

Kalanchoe (*Kalanchoe blossfeldiana*) is a qualitative short-day plant with a high aesthetic value. When the night length is less than a specified cultivar-dependent critical value, however, it does not develop flowers. This study investigated the effects of low-intensity supplementary or night interrupting (NI) blue (B) light on the plant performance and flower induction in kalanchoe ‘Rudak’. During the photoperiod in a closed-type plant factory with day/night temperatures of 23 °C/18 °C, white (W) LEDs were utilized to produce a photosynthetic photon flux density (PPFD) of 300 μmol m^−2^ s^−1^, and B LEDs were used to give supplementary/NI light at a PPFD of 10 μmol m^−2^ s^−1^. The control plants were exposed to a 10-h short day (SD, positive control) or a 13-h long day (LD, negative control) treatment without any B light. The B light was used for 4 h either (1) to supplement the W LEDs at the end of the SD (SD + 4B) and LD (LD + 4B), or (2) to provide night interruption (NI) in the SD (SD + NI-4B) and LD (LD + NI-4B). The LD + 4B and LD + NI-4B significantly enhanced plant growth and development, followed by the SD + 4B and SD + NI-4B treatments. In addition, the photosynthesis, physiological parameters, and activity of antioxidant systems were improved in those treatments. Except in the LD and LD + NI-4B, all plants flowered. It is noteworthy that kalanchoe ‘Rudak’ flowered in the LD + 4B treatment and induced the greatest number of flowers, followed by SD + NI-4B and SD + 4B. Plants grown in the LD + 4B treatment had the highest expression levels of certain monitored genes related to flowering. The results indicate that a 4-h supplementation of B light during the photoperiod in both the SD and LD treatments increased flower bud formation, promoted flowering, and enhanced plant performance. Kalanchoe ‘Rudak’ flowered especially well in the LD + 4B, presenting a possibility of practically inducing flowering in long-day seasons with B light application.

## 1. Introduction

Plants adapt to the information they acquire from the environment, such as the quality of light and adjust their biological cycles as a response [1]. Changes in light quality, which are influenced by the spectrum qualities of tissue pigments, have a significant impact on the anatomical, physiological, morphological, and biochemical aspects of leaves [2,3]. Plants’ growth and development are regulated by photoreceptors throughout their lives. Photoreceptors help plants time crucial developmental events like flowering and seed germination by monitoring the light environment [4]. Phytochromes and cryptochromes are two types of photoreceptors that allow plants to detect changes in the light quality [5,6]. Phytochrome is a photoreceptor that generally assimilates red (R) and far-red (Fr) light, while cryptochrome essentially absorbs ultraviolet-A (UV-A) and blue (B) light, and both photoreceptors assist in controlling the flowering [7]. Depending on the plant species, there may be many cryptochrome (CRY1 and CRY2) and phytochrome (PHYA, PHYB, PHYC, PHYD, and PHYE) variants [8,9].

Photoperiod modification can help minimize agricultural expenses by reducing production time and improving overall crop quality [10]. Seedlings and rooted cuttings are frequently supplemented with light to improve their quality, which takes the form of extra light that extends the day length, or supplemental light in the background of natural light [11]. NI uses lights to break up a lengthy period of darkness, resulting in modified long-day conditions [12,13]. During short-day seasons, NI efficiently expedited the flowering of long-day plants (LDPs) and allowed for earlier marketing or seed production, while long-day seasons delayed the flowering of short-day plants (SDPs) [14]. NI utilizing low-intensity light-emitting diodes (LEDs) should marginally influence the net photosynthesis and guarantee development advancement in tomatoes [15]. Low-intensity NI (3–5 μmol m^−2^ s^−1^ PPFD) was utilized to efficiently induce flowering and enhance growth rates in juvenile *Cymbidium aloifolium* [16].

Photosynthesis, chloroplast growth, chlorophyll creation, and plant chemical composition all benefit from B light [17]. According to studies, B light has a detrimental impact on stem elongation and results in a reduction of leaf area [18,19]. Senger [20] discovered that B light was important for chloroplast production and development, in addition to stomatal opening. Photoreceptors associated with B light may have a role in the flowering process, according to certain studies [21,22]. Supplementary B light stimulates the elongation of stems and internodes without affecting the flower bud development, according to Jeong et al. [23]. NI with B light did not successfully suppress flowering in the SDP chrysanthemum, even though B light is a visible light [24,25], despite results from our previous study that a 4-h B light supplementation during the photoperiod promoted flowering and increased the number of flower buds formed for chrysanthemum [26]. When cultivated in the SD and with 10 μmol m^−2^ s^−1^ PPFD NI-B, the SDP kalanchoe Spain flowered, however, the flowering of Lipstick was unaffected by the night interruption light (NIL) quality [27]. Plant responses to light quality are species-specific, according to research conducted under various light environments [17,28,29].

When plants are subjected to stress conditions such as light and chilling treatments, the antioxidant system comes into play to protect them. Plants have developed several endogenous antioxidant systems including non-enzymatic antioxidants (carotenoids, ascorbate, and tocopherol) and enzymatic antioxidants (superoxide dismutase (SOD), guaiacol peroxidase (GPX), catalase (CAT), etc.), which have evolved in plants to scavenge reactive oxygen species (ROS) [30]. Conversely, the activities of antioxidant enzymes in plants influenced by the light spectrum are more complicated and frequently described with contradictory findings. In reaction to the blue spectrum, Mastropasqua et al. [31] found the increased activity of the ascorbate peroxidase (APX) scavenger enzyme in detached oat leaves. B light supplementation improved the antioxidant activity of *Kalanchoe pinnata* [32]. Conversely, R and Fr light radiations were shown to be efficient in promoting antioxidant activity in pea seedlings and rice hulls [33,34]. These findings imply that various plant species have varied morphogenetic, photosynthetic, and antioxidant responses to light spectrum variations. Thus, it is necessary to investigate the suitable light quality for maximal biomass production and to evaluate different responses caused by varying light spectrums under artificial conditions.

Thus, we predicted that low-intensity B light supplied to either long-day or short-day conditions may promote SDP flowering. The effects of low-intensity (10 μmol m^−2^ s^−1^ PPFD) B light used as supplemental or NI light on photosynthesis, physiology, morphology, anatomy, antioxidant, and notably flowering and flowering-related gene expression in kalanchoe ‘Rudak’ were investigated in this study.

## 2. Materials and Methods

### 2.1. Plant Materials and Growth Conditions

The seedlings of kalanchoe (*Kalanchoe blossfeldiana* ‘Rudak’), a qualitative short-day plant (SDP), with 10 ± 2 leaves per plant were obtained from a commercial kalanchoe farm (J Flower, Gimhae, Korea) in early June of 2021. The seedlings were planted in a commercial medium (BVB Medium, Bas Van Buuren Substrates, EN-12580, De Lier, The Netherlands) in 10 cm plastic pots, and subsequently acclimated for one week on a greenhouse bench with sunlight and a natural photoperiod. After acclimation, for the subsequent photoperiodic light treatments, rooted cuttings were transferred to a closed-type plant factory (7700 cm long × 2500 cm wide × 2695 cm high, Green Industry Co. Ltd., Changwon, Korea) maintained at day 23 °C/night 18 °C with a relative humidity of 60% ± 10%. The closed-type plant factory was constructed such that numerous uniformly distributed holes allowed conditioned air to blow horizontally into the growing spaces. CO_2_ was supplemented from a compressed gas tank to maintain an atmospheric concentration of 350 ± 50 μmol∙mol^−1^. The kalanchoes were fertigated once a day (from 8:30 a.m. to 9:30 a.m.) throughout the experiment with a multipurpose nutrient solution (in mg∙L^−1^): 708.0 Ca(NO_3_)_2_∙4H_2_O, 246.0 MgSO_4_∙7H_2_O, 505.0 KNO_3_, 230.0 NH_4_H_2_PO_4_, 1.24 H_3_BO_3_, 0.12 CuSO_4_∙5H_2_O, 4.00 Fe-ethylene diamine tetra-acetic acid, 2.20 MnSO_4_∙4H_2_O, 0.08 H_2_MoO_4_, and 1.15 ZnSO_4_∙7H_2_O. Additionally, a three-replication randomized complete block design was employed with a total of 12 plants for each treatment, with 4 plants in each replication. Within a controlled environment, the photoperiodic light treatments were randomly located between replications to minimize the effects of light positioning.

### 2.2. Photoperiodic Light Treatments

**Figure 1:**
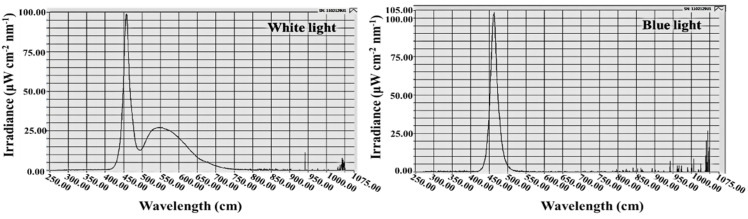
The spectral distribution of lights used in this experiment: The daily W light (~400–750 nm) provided by white LEDs and B light (450 nm) from blue LEDs used as the supplementary and night-interrupting light.

**Figure 2:**
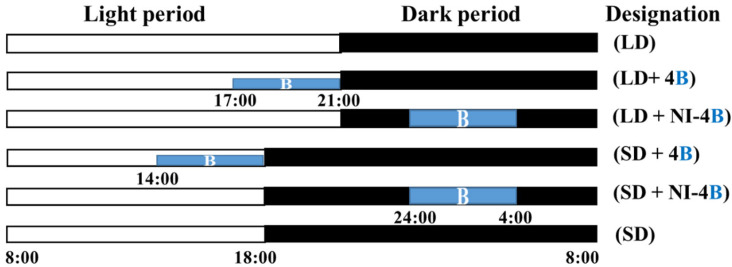
Supplementary and night-interrupting B light schemes employed in this study: The control plants were exposed to a 10-h short-day (SD, positive control) or 13-h long-day (LD, negative control) treatments without any B light. The B light was used for 4 h either (1) to supplement the W LEDs at the end of the SD (SD + 4B) and LD (LD + 4B) or (2) to provide night interruption (NI) in the SD (SD + NI-4B) and LD (LD + NI-4B). The light period began at 8:00 a.m., and the dark period ended at 8:00 a.m.

Plants were grown with a light intensity of 300 μmol·m^−2^·s^−1^ PPFD provided by white MEF50120 LEDs (More Electronics Co. Ltd., Changwon, Korea), B light at an intensity of 10 μmol·m^−2^·s^−1^ PPFD was provided by LEDs for the photoperiodic light treatments (Figure 1). Various photoperiods were used in this study. Plants in the control were grown with a 13-h long day treatment (LD) and a 10-h short day treatment (SD) without any B light. The lighting periods during the night interruption (referred to as “photoperiods” hereafter) were as follows: B light was used for 4 h either (1) to supplement the W LEDs at the end of the SD (SD + 4B) and LD (LD + 4B) or (2) to provide night interruption (NI) in the SD (SD + NI-4B) and LD (LD + NI-4B) (Figure 2). The average PPFD of each treatment was measured with a quantum radiation probe (FLA 623 PS, ALMEMO, Holzkirchen, Germany) 30 cm above the benchtop. The lighting was adjusted such that the same PPFD levels were provided to the plants regardless of the light treatment. A spectroradiometer (USB 2000 Fiber Optic Spectrometer, Ocean Optics Inc., Dunedin, FL, USA; detects wavelength between 200 to 1000 nm) was used to scan the spectral distribution 35 cm above the benchtop in 1 nm wavelength intervals. For each light treatment, the spectral distribution and characteristics were measured at three different locations within the plant growing areas.

### 2.3. Measurements of the Morphological Parameters

The plant growth parameters, as shown in Table 2, were collected after 60 days of the photoperiodic light treatments. All leaves with a length greater than 2 cm were counted to determine the number of leaves per plant. Divided samples of the shoot and root were dried at 70 °C for five days in a drying oven (Venticell-222, MMM Medcenter Einrichtungen GmbH., Munich, Germany) before the dry mass measurements were taken with an electronic scale (EW 220-3NM, Kern and Sohn GmbH., Balingen, Germany). The color values of the leaves and flowers were measured with a color reader CR-11 (1994 Minolta Co., Ltd. Osaka, Japan) and analyzed by “Munsell Color Palette System” https://pteromys.melonisland.net/munsell/, accessed on 12 August 2021, to obtain the corresponding color chart. In addition, the plants were harvested and put in liquid nitrogen immediately, then kept in a −80 °C refrigerator for the subsequent physiological investigations.

### 2.4. Leaf Anatomical Features and Chloroplast Distribution

In each treatment, six leaf segments (2 cm^2^) without midribs were collected from fully expanded leaves for the leaf cross-section anatomical observations. Firstly, fresh sections were fixed in formalin-acetic acid-alcohol (FAA) solution containing 50% (*v*/*v*) ethanol, 45% (*v*/*v*) paraformaldehyde, and 5% (*v*/*v*) glacial acetic acid at 4 °C for 24 h. Subsequently, the leaf samples were sliced to an appropriate thickness by the freehand slice method after being dehydrated in a graded series of ethanol solutions [95, 75, 50, 25, and 10% (*v*/*v*) ethanol] three times for each treatment for 40 min. The slices were mounted on glass slides and observed without staining using an optical microscope (ECLIPSE Ci-L, Nikon Corporation, Tokyo, Japan). ImageJ (ImageJ bundled with 64-bit Java 1.8.0_172, National Institutes of Health, Bethesda, MD, USA) was used to estimate the thickness of the leaves, palisades, and spongy tissues.

The chloroplast distribution was observed in the stomatal guard cells. Leaf pieces were fixed in a glutaric dialdehyde solution [4% (*v*/*v*) glutaraldehyde; 1% (*w*/*v*) osmium tetroxide], dehydrated in a graded series of acetone solutions, and embedded in Epon812 [35]. Subsequently, the ultrathin section was cut using the rotary microtome (RM2125RT, Leica, Nussloch, Germany) after uranyl acetate and lead citrate staining. A transmission electron microscope (H-600IV, TEM, Hitachi, Nagoya, Japan) was used for photographic examinations and measurements with ImageJ (ImageJ bundled with 64-bit Java 1.8.0_172, National Institutes of Health, Bethesda, MD, USA).

### 2.5. Stomatal Density and Morphological Characteristics

Six leaf replications were randomly selected from each treatment, and the lower epidermis of leaves without midribs in a similar position of fully expanded leaves were used to observe the stomatal morphology. The leaf samples were fixed for 48 h at 4 °C in a mixed solution containing 45% (*v*/*v*) acetone, 45% (*v*/*v*) ethanol, and 10% (*v*/*v*) distilled water. The stomata were observed by tearing the epidermis off the leaf with gummed tape. The excised samples were observed at different magnifications with an optical microscope (ECLIPSE Ci-L, Nikon Corporation, Tokyo, Japan) and analyzed with ImageJ (ImageJ bundled with 64-bit Java 1.8.0_172, National Institutes of Health, Bethesda, MD, USA). The stomatal density was determined by dividing the number of stomata by the area where the number of stomata was recorded. The morphological parameters of the stomata include the length and width of guard cell pairs in addition to stomatal pores measured by Sack and Buckley’s description [36].

### 2.6. Chlorophyll Content

0.1 g of fresh leaves were collected for testing the chlorophyll content (mg∙g^−1^), and six replicates were employed for each treatment. All samples were dipped in 10 mL of N,N-dimethyl formamide solution and left in the dark for 48 h at 4 °C before being tested for Chl a, b, and a + b. The absorbances of the supernatant at 645 and 663 nm were recorded with a UV spectrophotometer (Libra S22, Biochrom Ltd., Cambridge, UK). The chlorophyll content was estimated according to Sim et al. [37].

### 2.7. Measurements of Photosynthesis and Chlorophyll Fluorescence

The photosynthetic parameters, including the net photosynthetic rate (*P*_n_), transpiration rate (*T*_r_), stomatal conductance (*G*_s_), and intercellular CO_2_ concentration (*C*_i_) were measured on the youngest mature leaf of each plant with a leaf porometer (SC-1, Decagon Device Inc., Pullman, WA, USA). For this purpose, 3 cm^2^ of a terminal leaflet was enclosed in a leaf chamber mounted horizontally. From 9:30 to 11:30 a.m., these parameters were measured in the closed-type plant factory to keep the same steady conditions.

The miniaturized pulse-amplitude-modulated photosynthesis yield was used to detect the chlorophyll fluorescence. Each plant was moved to a dark chamber for 30 min to adapt before being measured with a photosystem (Fluor Pen FP 100, Photon Systems Instruments, PSI, Drásov, Czech Republic). The measurements involved the maximal PSII quantum yield (*F*v/*F*m), photochemical efficiency of PSII (*F*v′/*F*m′), and coefficient of photochemical quenching (*qP*). All parameters were calculated using the methods reported by Maxwell et al. [38].

### 2.8. Accumulation of Carbohydrates and Soluble Proteins

Well-mixed 0.3 g fresh leaf and stem samples were obtained at night and tested according to the Anthrone colorimetric method [39,40] for the starch and soluble sugar measurements. The method of soluble protein extraction was as follows: fresh leaves were collected, immediately immersed in liquid nitrogen, and ground into a fine powder over an ice bath. 0.1 g of the powder was homogenized in 50 mM PBS (1 mM EDTA, 1 mM polyvinylpyrrolidone, and 0.05% (*v*/*v*) triton-X, pH 7.0). The resulting mixture was then centrifuged (13,000 rpm, 4 °C, 20 min) to obtain the supernatant that would be used afterward for the total protein estimation and enzyme activity assay [41]. The total protein estimations were conducted using Bradford’s reagent [42,43]. In addition, the contents of soluble sugars, starch, and soluble proteins were measured with a UV spectrophotometer (Libra S22, Biochrom Ltd., Cambridge, UK) in A_630 nm_, A_485 nm_, and A_590 nm_, respectively.

### 2.9. Enzyme Activities

The protein obtained from the previous step was used to analyze the enzymatic activities. As described by Aeibi et al., the catalase (CAT) activity was measured in the homogenates by directly detecting the induction of H_2_O_2_ in A_240 nm_ [44]. The reaction medium contained 50 mM potassium phosphate buffer (PH 7.0), 10 mM H_2_O_2_, and 0.1 mL enzyme extract in a final volume of 3 mL at 25 °C. The extinction coefficient (40 mM^−1^ cm^−1^) for H_2_O_2_ was used to calculate the CAT activity. The activity of ascorbate peroxidase (APX) was measured for 1 min at A_290 nm_ (extinction coefficient 2.9 mM^−1^ cm^−1^) according to Nakano [45]. The reaction mixture contained 50 mM sodium phosphate buffer (PH 7.0), 0.1 mM EDTA, 1 mM ascorbate acid, 2.5 mM H_2_O_2_, and 50 μL of enzyme extract. Castillo et al. [46] proposed a method for measuring the guaiacol peroxidase activity (GPX). The 3 mL reaction mixture contained 10 mM guaiacol, 50 mM H_2_O_2_, 50 mM phosphate buffer (PH 6.0), and 50 μL enzyme extract. The extinction coefficient of 26.6 mM^−1^ cm^−1^ was used to compute the absorbance at A_470 nm_. The capacity of superoxide dismutase (SOD) to block the photochemical reduction of nitroblue tetrazolium (NBT) was measured as described by Becana et al. [47]. The 3 mL reaction mixture solution contained 50 mM potassium phosphate buffer (PH 7.8), 50 mM methionine, 75 μM NBT, 20 μM riboflavin, 0.1 mM EDTA, and 0.1 mL of enzyme extract. The reaction was performed for 15 min. Blanks and controls were run similarly to the other groups but without light and enzyme extracts, respectively. By monitoring at A_560 nm_, one unit of SOD was defined as the quantity of enzyme that provided a 50% inhibition of the NBT reduction.

The enzymatic activities of the key enzymes related to sucrose synthesis (SS, SPS, PEPC, and PEPP), starch synthesis (ADPGPPase, UDPGPPase, and SSS), and photosynthesis (Rubisco) were measured with a UV spectrophotometer (Libra S22, Biochrom Ltd., Cambridge, UK). The SS and SPS were determined in a 1 mL reaction mixture containing a 500 μL enzyme extract at 34 °C for 1 h. A 300 μL 30% (*v*/*v*) KOH was added to this mixture and was then placed in a water bath at 100 °C for 10 min, after which it was gradually cooled to room temperature. The mixture was subjected to incubation at 40 °C for 20 min after a 200 μL 0.15% (*v*/*v*) anthrone–sulfuric acid solution was applied and the enhancement of A_620 nm_ was monitored. The PEPC was assayed in a 1 mL reaction mixture consisting of 50 mM Tris-HCl (pH 8.0), 5 mM MnCl_2_, 2 mM DTT, 10 mM NaHCO_3_, 0.2 mM NADH, 5 unit NAD-MDH, and a 160 μL enzyme extract. The reaction was initiated by adding 2.5 mM phosphoenolpyruvate (PEP). The PEPP was determined in a 1.5 mL reaction mixture containing 100 mM imidazole-HCl (pH 7.5), 50 mM KCl, 10 mM MgCl_2_, 0.05% (*w*/*v*) BSA, 2 mM DTT, 150 μM NADH, 1 unit LDH, 2 mM ADP, and a 150 μL enzyme extract. The reaction was initiated with 2 mM PEP, and the increase in the A_412 nm_ was monitored. The RuBisCO total activity was measured by injecting 100 μL of the supernatant into 400 μL of an assay mixture consisting of 50 mM Tris-HCl (pH 8.0), 5 mM DTT, 10 mM MgCl_2_, 0.1 mM EDTA, and 20 mM NaH_14_CO_3_ (2.0 GBq mmol^−1^) at 30 °C. After a 5-min activation period, the reaction was initiated via the addition of RuBP to 0.5 mmol L^−1^ and was terminated after 30 s with 100 μL of 6 mol L^−1^ HCl. The above description of enzymatic activities was conducted in accordance with the directions provided by Feng et al. and Yang et al. [48,49]. Moreover, the activities of soluble starch synthase (SSS), adenosine diphosphate glucose pyro-phosphorylase (ADPGPPase), and uridine diphosphate glucose pyrophosphorylase (UDPGPPase) were measured according to the protocol described by Doehlert et al. and Liang et al. [50,51].

### 2.10. Real-Time Quantitative PCR Verification

Well-mixed 0.1 g fresh leaf, stem, and root samples were collected from kalanchoe plants for the gene expression analyses. All samples were immediately frozen in liquid nitrogen. The total RNA was extracted using an Easy-Spin total RNA extraction kit (iNtRON Biotechnology, Seoul, Korea). The total RNA was then used for first-stand cDNA synthesis with the GoScript Reverse Transcription System (Promega, Madison, WI, USA) according to the manufacturer’s protocols. Real-time quantitative PCR was conducted in a real-time PCR system (CFX96, Bio-Rad, Hercules, CA, USA). Reaction volumes (20 μL) contained 1 μL of cDNA, 1 μL of each amplification primer (10 μM), 10 μL of 2 × AMPIGENE qPCR Green Mix Lo-ROX (Enzo Life Sciences Inc., Farmingdale, NY, USA), and 7 μL ddH_2_O (double distilled water). The 2^−ΔΔCt^ method was used for the data analysis.

The expression level of key genes related to phytochromes (*KfPHYA* and *KfPHYB*), cryptochromes (*KfCRY1*), and flowering (*KfFT*, *KfFPF-1*, and *KfPEF-4*) were searched. The primers were designed through https://www.ncbi.nlm.nih.gov/tools/primer-blast/, accessed on 20 August 2021, according to the sequences which were obtained from https://phytozome-next.jgi.doe.gov/info/Kfedtschenkoi_v1_1, accessed on 20 August 2021. Moreover, *KfACTIN* was selected as a reference gene. All the details of target genes are listed in Table 1.

### 2.11. Statistical Analysis

Significant differences among the treatments were assessed by an analysis of variance (ANOVA), followed by Duncan’s multiple range test at a probability (*p*) ≤ 0.05 with a statistical program (SAS, Statistical Analysis System, V. 9.1, Cary, NC, USA). The differences between each treatment were tested by Student’s *t*-test (*p*) ≤ 0.05. Fisher’s least significant difference test was used for the *F*-test between treatments. Moreover, the experimental assays used to obtain all results were repeated six times and are presented as the mean ± standard error.

## 3. Results

### 3.1. Morphological Analyses

**Figure 3:**
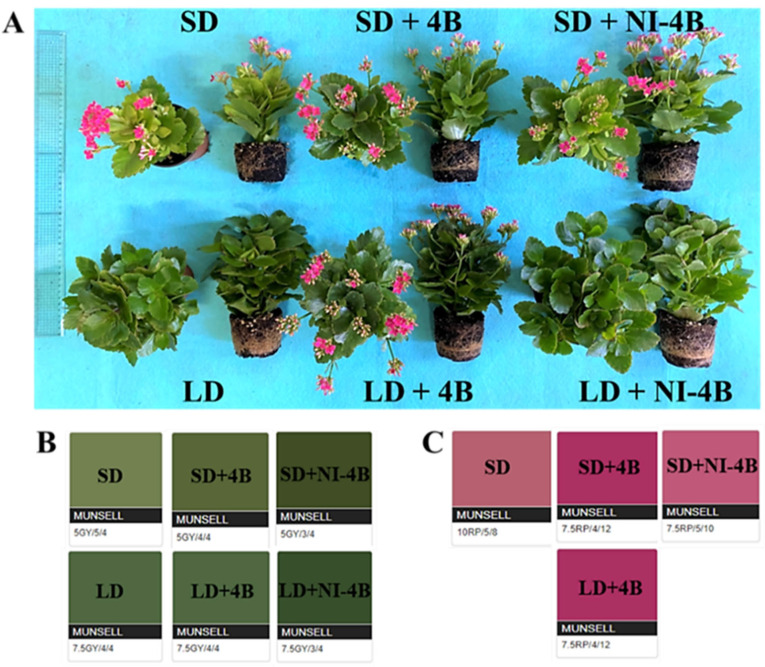
The effects of the supplementary and night-interrupting 10 μmol∙m^−2^∙s^−1^ PPFD B light on the flowering of kalanchoe (*Kalanchoe blossfeldiana* ‘Rudak’), after 60 days of exposure to the photoperiodic light treatments: The morphology (**A**); leaf color charts (**B**); and flower color charts (**C**) [The color charts of leaves and flowers were analyzed by ‘Munsell Colortell System’; e.g., in ‘5GY/5/4’, ‘5GY’ indicates ‘hue’, ‘5’ indicates ‘value’ (the larger the value, the lighter the color), and ‘4’ indicates ‘chroma’ (the larger the value, the brighter the color)] (See Figure 2 for details on the photoperiodic treatments with B light).

The photoperiodic light treatments with low-intensity supplementary or night-interrupting B light had significant effects on the morphological appearances of the kalanchoes (Figure 3 and Table 2). By comparing the plants to each other, “photoperiod” and “supplementary and night-interrupting B light” were two important growth factors for the development of kalanchoes. In both long-day and short-day conditions, the supplementary and night-interrupting B light treatments significantly increased the plant height, canopy diameter, stem diameter, number of nodes, and shoot fresh/dry weights. Plants in the LD + 4B, especially the LD + NI-4B, had greater growth and development parameters than those in the other treatments. Compared to short-day conditions, long-day conditions obviously promoted branching. Supplementary and night-interrupting B light applied in short-day conditions as well as LD + NI-4B showed a positive correlation with branching, while the decreased branching induced by LD + 4B may be replaced by inducing more flowers when compared with LD and LD + NI-4B treatments. Additionally, without any B light, LD treatment promoted the internode length more than SD did, while supplementary and night-interrupting B light significantly shortened the internode length of kalanchoes among all short-day and long-day conditions.

Furthermore, “long-day periods” and “B light” positively affected the leaf number. In addition, long-day increased the leaf size when compared to short-day conditions. However, supplementary and night-interrupting B light resulted in smaller leaves. LD + NI-4B resulted in the longest root length, followed by the LD + 4B treatment. Among all SD treatments, SD + 4B, and particularly the SD + NI-4B treatment, visibly improved the root fresh and dry weights, while the supplementary and night-interrupting B light did not induce such significant differences in long-day conditions, but the root fresh and dry weights were always greater in LD treatments than in SD treatments.

Most interestingly, LD + 4B induced flowering, resulting in the greatest number of flowers, but delayed flowering compared with all SD treatments. Moreover, SD treatments induced the earliest flowers but also the lowest number of flowers, while SD + 4B and SD + NI-4B markedly increased the number of flowers, which appeared late. However, there were no significant differences in the effects of SD + 4B and SD + NI-4B on flowering. The color of leaves and flowers also responded to the photoperiodic light treatments with low-intensity supplementary or night-interrupting B light. In short-day conditions, the leaf color in all three treatments was yellowish-green, and the color value went darker in SD + 4B, especially in SD + NI-4B, but the color chroma remained the same. With the same pattern as in SD + 4B and + NI-4B, LD + 4B and + NI-4B increased the color value but not the chroma, while the leaf color tended to be pure green, and the richest leaf color was observed in LD + NI-4B, followed by LD + 4B. Moreover, supplementary and night-interrupting B light also influenced the hue, value, and chroma of the flower color, and the brightest flowers were observed in SD + 4B and LD + 4B treatments.

### 3.2. Anatomical Features of Leaves

**Figure 4:**
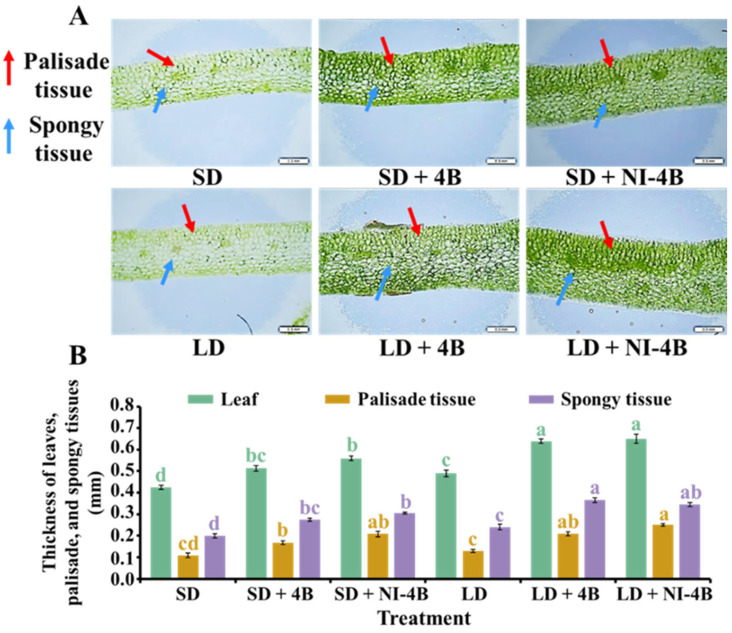
The effects of the supplementary and night-interrupting 10 μmol∙m^−2^∙s^−1^ PPFD B light on the anatomical features of kalanchoe (*Kalanchoe blossfeldiana* ‘Rudak’) leaves, after 60 days of exposure to the photoperiodic light treatments: the micrographs (**A**); and analysis charts (**B**) of leaf structures. Bars indicate 0.3 mm. Vertical bars indicate the means ± standard error (*n* = 6). Different lowercase letters indicate significant separation within treatments by the Duncan’s multiple range test at *p* ≤ 0.05. (See Figure 2 for details on the photoperiodic treatments with B light).

Leaf structure, such as leaf thickness, palisade tissue, and sponge tissue, affects the photosynthetic rate and showed certain differences under different light qualities (Figure 4). Compared to SD and LD treatments, kalanchoe leaves were significantly thicker after applying supplementary and night-interrupting B light treatments. The thicker leaves were generally observed in long-day conditions, and LD + 4B and LD + NI-4B resulted in the greatest leaf thicknesses. In addition, the supplementary and night-interrupting B light applied in both long-day and short-day conditions improved the development of palisade and spongy tissues and induced neatly arranged columnar palisade tissue cells. Furthermore, the spongy tissue consisting of more layers of irregularly distributed cells were better developed. Moreover, from our results, the long-day conditions were more beneficial for natural leaf growth. Taken together, LD + 4B and LD + NI-4B resulted in the greatest leaf structure growth and development.

### 3.3. Morphological Characteristics of the Stomata

**Figure 5:**
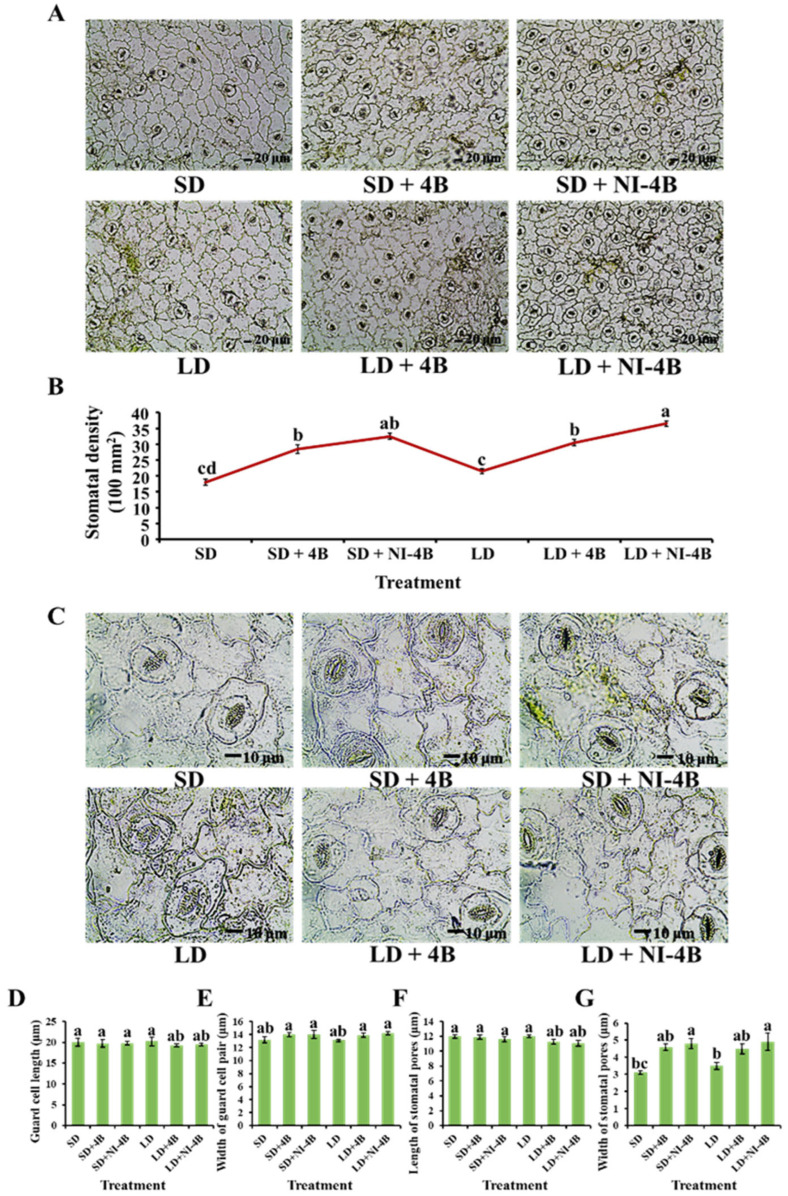
The effects of the supplementary and night-interrupting 10 μmol∙m^−2^∙s^−1^ PPFD B light on the stomatal parameters of kalanchoe (*Kalanchoe blossfeldiana* ‘Rudak’) leaves, after 60 days of exposure to the photoperiodic light treatments: The stomatal density (**A**,**B**) (10× enlargements); the length and width of guard cells and stomatal pores (**C**–**G**) (40× enlargements). Bars indicate 10 μm. Vertical bars indicate the means ± standard error (*n* = 6). Different lowercase letters indicate significant separation within treatments by Duncan’s multiple range test at *p* ≤ 0.05. (See Figure 2 for details on the photoperiodic treatments with B light).

Stomata is the main channel and regulatory organ for plants to exchange water and gas with the environment, which responds to changes in the environmental factors and regulates the balance between carbon assimilation and water loss in plants. Stomatal density and morphological characteristics strongly responded to supplementary and night-interrupting B light when plants were exposed to the various photoperiodic light treatments (Figure 5). In both short-day and long-day conditions, supplementary (particularly the night-interrupting) B light notably increased the stomatal density and promoted stomatal opening, while there were no significant differences in the stomatal size among all six treatments. Overall, the significantly increased stomatal density and opening under supplementary and night-interrupting B light positively affected the CO_2_ absorption capacity, thus further improving the photosynthesis of kalanchoe plants.

### 3.4. Chloroplast Distribution and Chlorophyll Content

**Figure 6:**
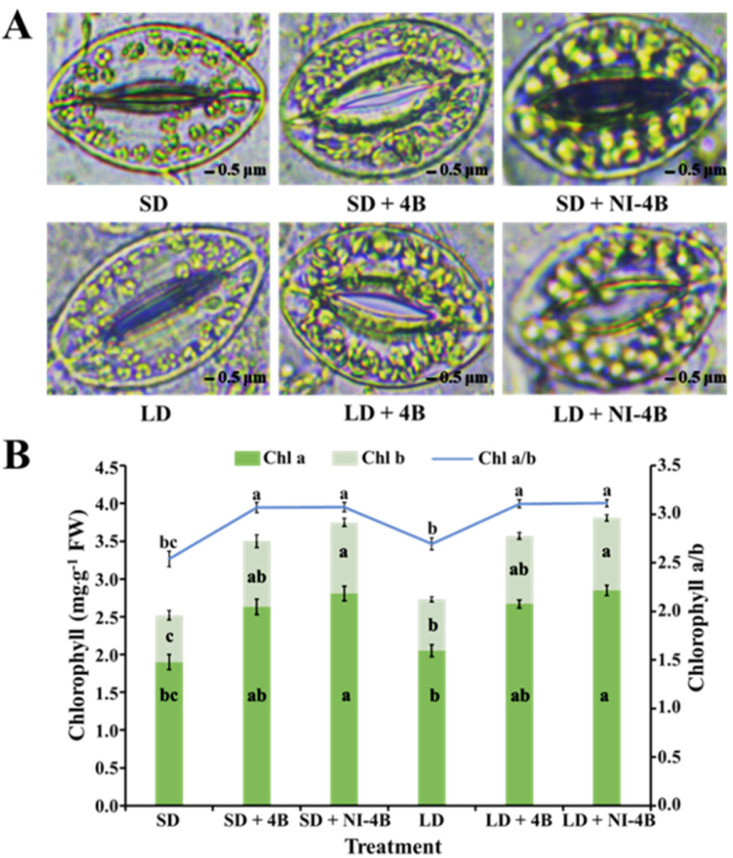
The effects of the supplementary and night-interrupting 10 μmol∙m^−2^∙s^−1^ PPFD B light on the chloroplast development and distribution (**A**); and chlorophyll content (**B**) of kalanchoe (*Kalanchoe blossfeldiana* ‘Rudak’) leaves, after 60 days of exposure to the photoperiodic light treatments; bars indicate 0.5 μm. Vertical bars indicate the means ± standard error (*n* = 6). Different lowercase letters indicate significant separation within treatments by Duncan’s multiple range test at *p* ≤ 0.05. (See Figure 2 for details on the photoperiodic treatments with B light).

Chloroplasts are important organelles for plant energy conversion and photosynthesis. Compared to SD and LD treatments, SD/LD + 4B, and particularly the SD/LD + NI-4B treatments, prominently increased the number and distribution of chloroplasts in the guard cells of kalanchoe leaves (Figure 6A). Our results showed that supplementary and night-interrupting B light were beneficial to the development of the chloroplast and induced an abundance of uniformly distributed chloroplasts that are regular, full, and smooth in shape. The chlorophyll content was an important substance in plant primary production and can indirectly reflect the vegetation’s health status and photosynthetic capacity. B light was beneficial to the synthesis of chlorophylls in kalanchoe (Figure 6B). The same pattern as chloroplast development was shown, where SD/LD + 4B and particularly SD/LD + NI-4B significantly enhanced the chlorophyll content and chlorophyll a/b. These influences induced by supplementary or night-interrupting B light all positively affect photosynthesis.

### 3.5. Photosynthetic and Chlorophyll Fluorescence Characteristics

In our study, the *P*n of kalanchoe plants was significantly lower when grown under short-day conditions than under long-day conditions, especially under the SD treatment. The applied supplementary and night-interrupting B light in both long-day and short-day conditions obviously increased the *P*n. This promotion was more obvious in long-day conditions, and the LD + NI-4B treatment resulted in the greatest *P*n (Table 3). Moreover, the changing trend of other photosynthetic characteristics (*T*r, *G*s, and *C*i) was the same as that of the stomatal density and opening pattern (Figure 5), where the LD/SD + NI-4B treatments induced the greatest values, followed by LD/SD + 4B, and SD resulted in the lowest values (Table 3).

Chlorophyll fluorescence is another element that defines photosynthetic activity. It provides information on PSII activity in response to various environmental conditions. Furthermore, because of PSII’s functional interaction with the other components of the photosynthetic apparatus, chlorophyll fluorescence may be used as an indirect indicator of the state of the integral photosynthetic process. Consistent with the chlorophyll content changing pattern (Figure 6), the chlorophyll fluorescence (*F*v/*F*m and *F*v′/*F*m′) in dark-adapted leaves varied dramatically in response to applied supplementary and night-interrupting B light; however, there was nothing that responded to the photoperiod (short-day or long-day conditions) (Table 3). The enhanced PSII activity and photosynthetic efficiency were observed in LD/SD + 4B, and especially in LD/SD + NI-4B. This may be an important factor in that supplementary and night-interrupting B light caused biomass increase (Table 2).

### 3.6. Accumulation of Carbohydrates and Soluble Proteins

**Figure 7:**
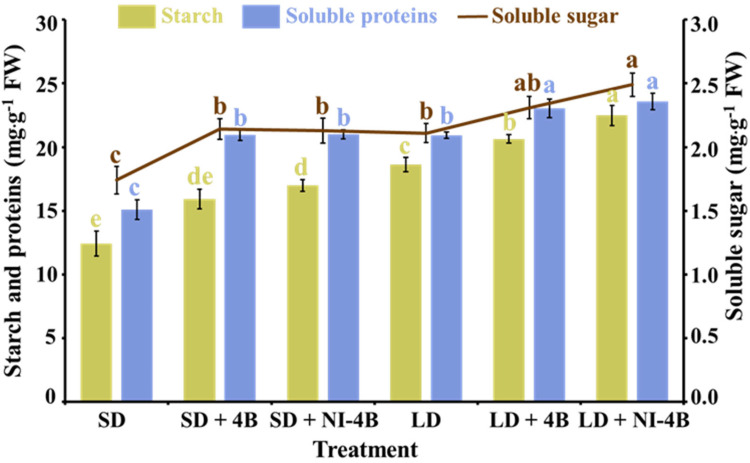
The effects of the supplementary and night-interrupting 10 μmol∙m^−2^∙s^−1^ PPFD B light on the accumulation of carbohydrates and soluble proteins in kalanchoe (*Kalanchoe blossfeldiana* ‘Rudak’), after 60 days of exposure to the photoperiodic light treatments. Vertical bars indicate the means ± standard error (*n* = 6). Different lowercase letters indicate significant separation within treatments by the Duncan’s multiple range test at *p* ≤ 0.05. (See Figure 2 for details on the photoperiodic treatments with B light).

Results of accumulation of carbohydrates and soluble proteins showed a strong effect of the supplementary and night-interrupting B light employed in the photoperiodic light treatments (Figure 7). In both short-day and long-day conditions, supplementary and night-interrupting B light applications significantly promoted the accumulation of carbohydrates and soluble proteins, and this positive effect was more obvious in long-day conditions, especially in the LD + 4B and LD + NI-4B treatments, which is consistent with the biomass changing tendency (Table 2). Overall, the well-developed leaf structures (Figure 4), outstanding stomatal characteristics (Figure 5), splendid number and distribution of chloroplasts (Figure 6A), and great chlorophyll content (Figure 6B) were induced by supplementary and night-interrupting B light in both short-day and long-day conditions, leading to excellent photosynthesis, and further promoted the accumulation of organic nutrients which can be viewed as an indicator reflecting the healthy development state of plants.

### 3.7. Enzymatic Activities

**Figure 8:**
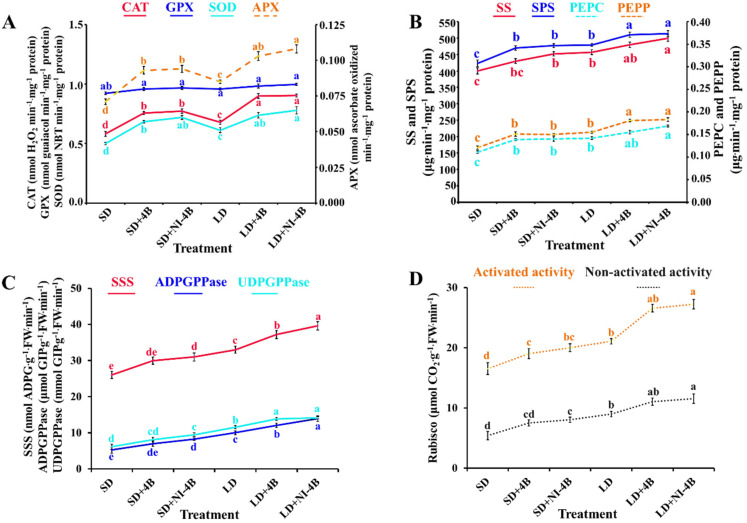
The effects of the supplementary and night-interrupting 10 μmol∙m^−2^∙s^−1^ PPFD B light on the enzymatic activities in kalanchoe (*Kalanchoe blossfeldiana* ‘Rudak’), after 60 days of exposure to the photoperiodic light treatments: the reactive oxygen species (ROS) scavenging enzymatic activities (**A**): catalase (CAT), guaiacol peroxidase (GPX), superoxide peroxidase (SOD), and ascorbate peroxidase (APX); sucrose synthesis enzymatic activities; (**B**): sucrose synthase (SS), sucrose phosphate synthase (SPS), phosphoenolpyruvate carboxykinase (PEPC), and phosphoenolpyruvate phosphatase (PEPP); starch synthesis enzymatic activities; (**C**): soluble starch synthase (SSS), adenosine diphosphate glucose pyro-phosphorylase (ADPGPPase), uridine diphosphate glucose pyro-phosphorylase (UDGPPase); photosynthesis enzymatic activities; (**D**): activated and non-activated activity of RuBisCo. Vertical bars indicate the means ± standard error (*n* = 6). Different lowercase letters indicate significant separation within treatments by the Duncan’s multiple range test at *p* ≤ 0.05. (See Figure 2 for details on the photoperiodic treatments with B light).

In our study, with the comparison of supplementary and night-interrupting B light applied in various photoperiodic treatments, significant differences of some key enzymes related to plant physiology were observed (Figure 8). There were four reactive oxygen species (ROS)-scavenging enzymes (CAT, GPX, SOD, and APX) investigated (Figure 8A). Applied supplementary and night-interrupting B light in both short-day and long-day conditions raised the activities of CAT, SOD, and APX. These promotion effects were more obvious in long-day conditions, and those in LD + 4B/NI-4B were always the greatest, while the least significant effects were induced by SD, followed by LD. These results showed that the antioxidant system of B light-treated plants increased antioxidant enzymes to balance the damage of peroxidation of plant cells. In contrast, the activity of GPX was especially stable, and nothing responded to supplementary and night-interrupting B light applied in both short-day and long-day conditions.

Moreover, enzymes related to carbohydrate synthesis [sucrose synthesis (SS, SPS, PEPC, and PEPP) and starch synthesis (SSS, ADPGPPase, and UDGPPase)] also positively changed in response to the photoperiodic treatments with supplementary and night-interrupting B light (Figure 8B,C). Roughly the same trend was observed as follows: LD + NI-4B ≥ LD + 4B > LD ≥ SD + NI-4B ≥ SD + 4B > SD. In addition, the activity of RuBisCo (both activated and non-activated), which is the key enzyme for CO_2_ fixation in photosynthesis, showed nearly the same pattern as *P*n did (Figure 8D and Table 3), where supplementary and night-interrupting B light applied in the photoperiodic treatments significantly improved the enzymatic activity of RuBisCo, especially in LD + 4B/NI-4B. Taken together, these results strongly proved that the application of B light markedly improved plant health and vitality, and at the same time, it also can be used as an important basis for the greater biomass and high photosynthetic efficiency in B light-treated plants.

### 3.8. Gene Expressions

**Figure 9:**
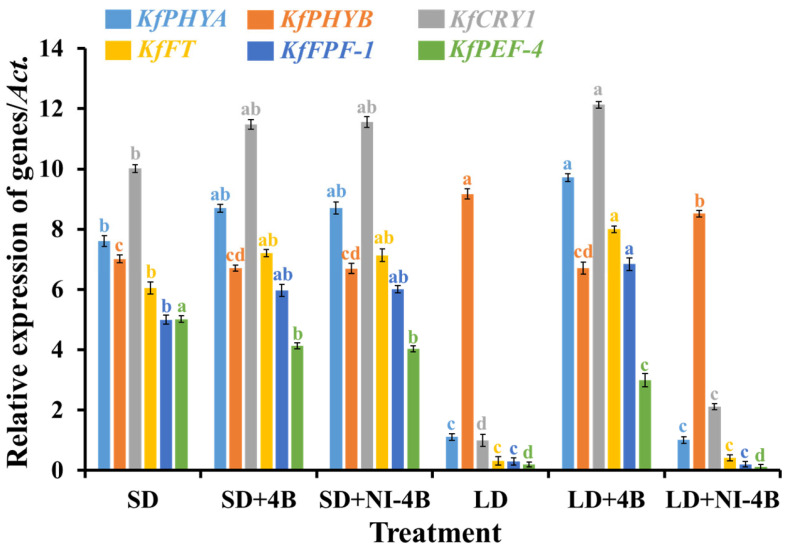
The effects of the supplementary and night-interrupting 10 μmol∙m^−2^∙s^−1^ PPFD B light on the gene expression levels in kalanchoe (*Kalanchoe blossfeldiana* ‘Rudak’), after 60 days of exposure to the photoperiodic light treatments. Vertical bars indicate the means ± standard error (*n* = 6). Different lowercase letters indicate significant separation within treatments by the Duncan’s multiple range test at *p* ≤ 0.05. (See Figure 2 for details on the photoperiodic treatments with B light; see Table 1 for details of genes).

Flowering is the transition from vegetative to reproductive growth. To validate the differential expression of the specific genes related to the flowering pathway, quantitative (q) RTPCR analyses were performed for the key genes of interest (Figure 9). The flowering promoter genes (*KfPHYA*, *KfCRY1*, *KfFT*, and *KfFPF-1*) were expressed following the same pattern in all six treatments: LD + 4B > SD + NI-4B = SD + 4B > SD > LD + NI-4B ≥ LD. *KfPEF-4* was an early flowering promoter gene and greatly expressed in plants that were grown in SD, followed by SD + 4B/NI-4B, while extremely low expression levels were observed in LD and LD + NI-4B. In addition, LD + 4B resulted in the latest flowering but the greatest number of flowers. In contrast, the profuse expression of the flowering suppressor gene (*KfHYB*) was shown in LD, followed by LD + NI-4B, while a lower expression was observed in flowered treatments (SD, SD + 4B, SD + NI-4B, and LD + 4B). A comprehensive analysis of the above results revealed that the expression patterns of these flowering-related genes were in accordance with the flowering phenotypes, that LD + 4B induced the greatest number of late-blooming flowers, and there was no flowering formatted in both LD and LD + NI-4B (Figure 3 and Table 2).

## 4. Discussion

### 4.1. Growth and Morphology of Kalanchoes Were Influenced by Low-Intensity Supplementary and Night-Interrupting Blue Light in the Photoperiodic Light Treatments

Photomorphogenesis is a plant adaptation mechanism that allows plants to adapt to their surroundings. This interaction is straightforwardly affected by light, which is directed by light receptors, for example, cryptocryptochromes (CRY), phototropins (PHOT) that are sensitive to B light, and phytochromes (PHY), which are delicate to R light. Physiological and metabolic changes in distinct development pathways are induced by the signals provided by light receptors [52].

#### 4.1.1. Low-Intensity Supplementary and Night-Interrupting Blue Light in Photoperiodic Light Treatments Synthetically Affected the Development of Plant Shoots

In general, increasing the share of blue (B) light in the presence of white (W) light induces node formation, shortens the internodes, shrinks the leaf area, reduces the relative plant height, and increases the nitrogen/carbon (N/C) ratio [53]. In our study, the number of nodes increased but the internode length decreased under supplementary and night-interrupting B light treatments when compared to kalanchoe in SD and LD, respectively (Figure 3 and Table 2). Light quality, as an important characteristic of light environment, directly or indirectly affects the synthesis and transport of plant excitement. Irradiation of red (R) and B light at the seedling stage could significantly promote the growth of vegetable seedlings and increase the seedling index [54]. Different wavelengths of light can regulate the internode growth of plants by affecting the hormone levels in plants, and phytochromes can regulate the hypocotyl elongation and growth by affecting the endogenous gibberellic acid (GA) level of cowpea seedlings [55]. B light can increase the activity of auxin oxidase, which reduces the level of auxin in plants, weakens apical dominance, and enhances tillering ability, and thus inhibits internode elongation [56]. Hypocotyl elongation was correlated with different wavelengths of light. W light and B light inhibited stem elongation, while green (G) light significantly promoted internode elongation [54]. Exogenous application of indole acetic acid (IAA) or GA can restore hypocotyl elongation of lettuce seedlings inhibited by B light to a certain extent, suggesting that B light may inhibit hypocotyl elongation by reducing the endogenous GA levels of lettuce seedlings [57,58]. When the proportion of B light (R/B = 7:3) was appropriately increased in the light treatment, the height of seedlings decreased significantly, and the seedling strength index increased significantly [59].

However, from our results, the supplementary and night-interrupting B light promoted plant height in both short-day and long-day conditions, and the plants grown in long-day conditions always had a greater height than those in short-day conditions. This may be because different species respond differently to the light quality, and long-day photoperiods provide sufficient light for plant growth. Furthermore, plants in LD + 4B had the highest levels of *PHYA* and *CRY1* expression, whereas plants in LD + NI + 4B had the greatest level of *PHYB* expression. These two treatments also produced the tallest plants. Cryptochromes and phytochromes have been shown to impact the height of chrysanthemums [60]. In Arabidopsis, elevated *PHYB* levels can boost the expression of *AtGAox2*, a gene that regulates GA production [61]. Furthermore, both phytochromes and cryptochromes have been found to have a role in the control of plant hormone GA levels [61,62]. In this way, it is hypothesized that the high articulation levels of *PHYA*, *PHYB*, and *CRY1* found in plants filled in LD + 4B and LD + NI + 4B may advance the synthesis of GAs and finally result in greater plant height.

From the research of “The effect of different wavelength lights on photosynthesis of *Siraitia grosvenorii*, the plants cultured under B light with 14 μmol·m^−2^·s^−1^ PPFD light intensity had the best appearance, with bright green, large, and uniform leaves, moderate plant height, thick stems, and more lateral buds [63]. This indicates that B light promotes the lateral stretching of plants and increases the canopy diameter, which is consistent with our results (Figure 3 and Table 2). Moreover, B light increased the stem diameter and the plant biomass [64]. In our study, the stem diameter, shoot fresh weight, and dry weight all significantly improved with supplementary and night-interrupting B light in periodic light treatments (Table 2). Overall, B light can positively affect the accumulation of secondary metabolites in plants and benefit plant growth and development.

#### 4.1.2. Low-Intensity Supplementary and Night-Interrupting Blue Light in Photoperiodic Light Treatments Increased the Leaf Number and Decreased the Leaf Size

The leaf is the main organ for photosynthesis in plants and directly affects the photosynthetic capacity of plants, further affecting plant growth and development. In our study, a greater number of leaves with smaller sizes occurred in kalanchoes that were grown with photoperiodic treatments with supplementary and night-interrupting B light (Figure 3 and Table 2). Consistent with our results, Gautier et al. found that by increasing the proportion of B light, the petiole length of clovers could be decreased while the leaves became smaller, thus proving that the B light has an inhibitory effect on leaf growth [65]. Besides, compared with neutral film, the cultivation with a blue film cover significantly reduced the area of strawberry leaves [66]. This is because B light can increase the activity of IAA oxidase, decrease the IAA content, and inhibit plant elongation [67].

#### 4.1.3. Low-Intensity Supplementary and Night-Interrupting Blue Light in Photoperiodic Light Treatments Affected the Flower Color, Delayed the Formation of Flower Buds, and Improved the Number of Inflorescences by Controlling Flowering-Related Genes

Consistent with our previous study, the short-day plant chrysanthemum flowered in LD + 4B and LD + NI-4B treatments but took a longer time to bloom when compared with SD + 4B and SD + NI-4B treatments [26]. Kalanchoe, a qualitative short-day plant, flowered in LD + 4B and resulted in the greatest flower number (Figure 3 and Table 2). The flowering process is widely known to involve photoreceptors associated with B light [21,23]. Both CRY1 and CRY2 mediate the enhancement of flowering by B light [68]. In *Arabidopsis*, PHYA mediates flowering enhancement by far-red (FR) light, while PHYB mediates flowering suppression by red (R) light [69,70,71]. Although PHYA and PHYB are R light receptors, they have also been demonstrated to work in *Arabidopsis* under B light [72]. It has also been established that either PHYA or PHYB, in addition to cryptochromes, are necessary for B light responses [69,73]. The number of flowers per plant increased with the B light treatments in this study. This might be due to the high levels of CRY1 expression. Similarly, Park et al. [13] reported that during the NI, the light shifting from B resulted in a higher number of flowers per plant, which might be due to a high light energy induction as well as shadow avoidance reactions, in which plants stretch their internodes to escape the darkness. The flowering period in rice was slowed by NI with B light, but this delay was not replicated in the *phyb-1* mutant [74], indicating that PHYB is a negative regulator of flowering time. Even though chrysanthemum is a qualitative SD plant, those in the LD13 + 4B treatment flowered. This suggests that high levels of PHYA and CRY1 expression may cause flowering. However, further study is required to confirm this hypothesis.

In addition, in light quality stress environments, the flowering-related genes, such as FT (flowering locus T), FLC (flowering locus C), CO (constans), and PFT1 (phytochrome and flowering time 1), etc. participate in flowering regulation. Many studies have shown that FT gene expression is upregulated when plants are exposed to far-red light [75,76,77,78]. However, as the FT gene is located downstream of the flowering pathway and regulated by multiple pathways, its expression is upregulated when flowering is accelerated. *Arabidopsis* flowering accelerated when R∶FR decreased, but the Bla-6 ecotype did not show significant early flowering at low R∶FR, mainly because a high level of FLC prevented the promotion of flowering at low R∶FR by inhibiting the expression of FT [79]. Kim et al. [80] studied the promoting effect of FR light on flowering in *Arabidopsis* and found that the regulation of the light quality on CO may play an important role in the regulation of the flowering time in the natural environments. Later studies found that PFT1 responded to changes in the light quality and promoted plant flowering through two mechanisms, dependence on CO and dependence on CO directly acting on FT [81,82]. Rice flavin mononucleotide-binding protein OsHAL3 is a B light-sensing factor, which can interact with Hd1 and bind to the promoter of Hd3a in darkness. W light or B light can inhibit the interaction between OsHAL3 and Hd1 and delay flowering [83].

Moreover, anthocyanin decides the color of flowers and has attracted wide attention by its important value on ornamental plants. Flowers with more bright colors were observed in SD + NI-4B, especially in SD + 4B and LD + 4B (Figure 3). Longer illumination time, stronger illumination intensity, and B light are favorable and effective to the biosynthesis, stabilization, and accumulation of anthocyanins in most plants [84].

### 4.2. Photosynthesis, Physiology, and Internal Structures of Kalanchoes Were Influenced by Low-Intensity Supplementary and Night-Interrupting Blue Light in Photoperiodic Light Treatments

#### 4.2.1. Low-Intensity Supplementary and Night-Interrupting Blue Light in Photoperiodic Light Treatments Enhanced Leaf Structures, Chloroplast Distribution, and Chlorophyll Accumulation in Kalanchoes

The leaf structure parameters, such as leaf thickness, palisade tissues, spongy tissues, etc. affected the photosynthetic rate. The leaf structure also displayed differences in response to different light qualities. The supplementary and night-interrupting B light applied in photoperiodic light treatments significantly improved leaf development (Figure 4). Our results were consistent with the report that the B light enhanced the leaf thickness of *F. benjamina* and resulted in a larger rise in palisade parenchyma [85]. Other studies have found that the area of leaf palisade tissue, spongy tissue, and functional chloroplast of birch seedlings growing under B light is larger than those under W light and R light [86].

Chloroplasts are important organelles for plant energy conversion and photosynthesis, and their formation is regulated by the shape of the palisade cells. As in our study, well-developed chloroplasts were evenly distributed in guard cells of closely arranged, narrow, and continuous palisade tissues (Figure 6A), which can reduce the “sieve effect” by reducing the amount of light quantum-penetrating leaves [87]. Chloroplast movement through the direction consistent with the distribution of light quantum in the leaf (light gradient) enables it to make a full use of the light quantum penetrating the leaf so that photosynthesis is perfected as much as possible [88].

Chlorophylls (Chls) are the principal photosynthetic pigment in plants, and they play a role in plant physiological activities such as light-harvesting, photo-oxidation, and plant coloration, in addition to providing nutritional value as a precursor to essential vitamins and antioxidants [89]. The light spectra have a big role in photosynthetic pigment synthesis [90]. All of this demonstrates the significance of light quality in plant species phytochemical concentration optimization [91]. Plants cultivated under B light generated higher levels of Chl a, b, and Chl a + b, as well as a higher Chl a/b ratio (Figure 6B). B light has been shown to stimulate the biosynthesis of 5-aminolevulinic acid (ALA), a main Chl substrate [92]. By raising the Chl content and the Chl a/b ratio, B light simulates chloroplast development and improves photosynthetic efficiency [52,89]. Cucumber [17], cabbage seedlings [93], non-heading Chinese cabbage [94], lettuce, tomato, radish, pepper [95], and basil [96] have all been shown to benefit from B light in relation to increasing Chl content.

#### 4.2.2. Low-Intensity Supplementary and Night-Interrupting Blue Light in Photoperiodic Light Treatments Induced Excellent Stomatal, Photosynthetic, and Chlorophyll Fluorescence Characteristics

Stomata become the pathway of air and water in gas metabolisms such as carbon assimilation, respiration, and transpiration, which is of a great physiological significance. Stomatal characteristics strongly responded to the various light qualities. As shown in our study, supplementary and night-interrupting B light applied in photoperiodic treatments notably increased the stomatal density and promoted stomatal opening (Figure 5). In existing research reports, B light stimulates stomatal opening by suppressing anion release from the guard cells [97].

In our study, the *P*n of kalanchoes was significantly higher when grown under supplementary and night-interrupting B light treatments, especially in long-day conditions (Table 3). This is consistent with cucumber and *Acacia mangium* seedling outcomes [98,99]. Our findings also demonstrate that *P*n has a strong relationship with Chl content and RuBisCO activity. Moreover, the *T*r, *G*s, and *C*i positively responded to B light (Table 3). It is probable that the stomata of the seedlings exposed to B light were well-developed, suggesting benefits to the exchange of water and gas with the external environment, and the nitrogen accumulation of the B light-treated plants was higher than that of the plants under 100% W light treatments [100].

The Chl fluorescence of green plants mirrors their photosynthetic potential in a technical way [101]; some of the light absorbed by green plants is utilized for photosynthesis, while some is re-emitted as Chl fluorescence, and some is used for heat dissipation [102]. *F*v/*F*m and *F*v’/*F*m’ are parameters for the greatest photochemical efficiency of PSII, as well as the proportion of response focuses in PSII that are oxidized (open), which are signs of the photosynthetic efficiency. Among the supplementary and night-interrupting B light treatments used in this study, the *F*v/*F*m and *F*v’/*F*m’ values were promoted by B light (Table 3). Therefore, we hypothesize that the B light enhanced the rate of photosynthesis in kalanchoe plants by improving PSII activity. B light benefits kalanchoes because it enhances the efficiency of light energy conversion and allows for more energy accumulation for carbon assimilation in dark processes (Figure 7). Phalaenopsis showed lower *F*v/*F*m ratios in 100%R (0% B) light than in B light treatments in similar research [103].

#### 4.2.3. Low-Intensity Supplementary and Night-Interrupting Blue Light in Photoperiodic Light Treatments Directed the Accumulation of Carbohydrates and Proteins in Kalanchoes

Greater accumulation of carbohydrates and soluble proteins (both in leaves and stems) was detected in the treatments containing the supplementary and night-interrupting B light (Figure 7). These findings suggest that the B light stimulated the storage of organic materials in plant shoots by enhancing the accumulation of carbs and proteins. Under B light, cucumber, tomato, and sweet pepper transplants exhibited a rise in fresh and dry weights, as well as biomass accumulation, which is consistent with this study. They found that when all spectra were mixed with more B light, the FW of transplants increased [104]. According to Saebo et al. [86], a larger biomass accumulation under B light is attributed to an increase in starch content, which promotes photosynthate translocation out of the leaves, positively affecting the growth of other plant parts, such as flowers.

#### 4.2.4. Low-Intensity Supplementary and Night-Interrupting Blue Light in Photoperiodic Light Treatments Observably Activated the Enzymatic Activities in Kalanchoes

B light has been shown to increase antioxidant capacity in a variety of plant species [91,105]. It has been proposed that the increased antioxidant capacity of plants cultivated with higher B light ratios is related to the high concentration of pigments such as chlorophyll [91,106]. The first line of defense is superoxide dismutase (SOD), which transforms free radical oxygen into H_2_O_2_, which is subsequently transformed into H_2_O by CAT and APX. Accordingly, CAT and APX have the same activity [107]. Plant responses to biotic stressors are modulated by B light, which is a signal [108]. In the current study, higher activity of APX, CAT, and SOD was detected in plants grown under treatments with low-intensity supplementary and night-interrupting B light, while this lighting environment did nothing to affect the GPX activities (Figure 8A). Other research has found that the B light has a favorable effect on ascorbic acid production [105,109,110,111]. In several plant species, such as carnation [105], lettuce [111,112], *Rehmannia glutinosa*, and *Stevia rebaudiana* [113], it has been reported that the B light stimulates the activity of CAT and APX. These studies demonstrate that the B light has a beneficial influence on plant antioxidant capacity.

The RuBisCo, going about as RuBPCase, is a critical chemical in the Calvin cycle, the idea of which decides the photosynthetic effectiveness and efficiency. Light has been demonstrated to alter the activity of RuBPCase in algae in previous research [114,115]. The high photosynthetic rate caused by B light in our study might be linked to the plants’ high Chl content, light energy conversion efficiency, and RuBPCase activity (Figure 8D). In addition, supplementary and night-interrupting B light treatments activated the carbohydrate synthesis-related enzymes (Figure 8B,C), which may be caused by the increased chlorophyll content, PSII reaction center, and light-trapping complex [116]. Moreover, B light not only activates many enzymes related to carbohydrate degradation, photosynthetic carbon assimilation, photorespiration, and chlorophyll synthesis pathway, but also induces uridine diphosphate glucose (UDPG) and phosphoenolpyruvate carboxykinase (PEPC) [117], enhancing the phosphorglyceraldehyde dehydrogenase (PGD) capacity of RuBisCo and nicotinamide adenine dinucleotide phosphate (NADP) [118]. Thus, the activated carbohydrate metabolism helped promote the accumulation of starch and soluble sugars in B light-treated plants (Figure 7).

## 5. Conclusions

In summary, B light resulted in greater photosynthesis and physiological performances, promoted carbon assimilation and nitrogen absorption, accelerated material accumulations, met plant nutrient requirements, and induced expression of flowering-related genes. In particular, it stimulated antioxidant systems such as ROS-scavenging enzymatic activities that remove active oxides from plant cells and maintain homeostasis, thus promoting flowering in kalanchoe. Our results illustrate that the 4-h B light supplementation during the photoperiod induced a greater flower bud formation and promoted flowering, especially in the long-day conditions. Therefore, B light supplementation may be the most effective technique for inducing flowering, and it may be used in the commercial production of SD plants. Especially, this study presents a possibility of practically inducing SD plant flowering in long-day seasons by B light application.

## Figures and Tables

**Table 1:** List of the primers used to quantify the gene expression levels.

Name	Gene Identifier	Description	Forward Primer (5′ to 3′)	Reverse Primer (5′ to 3′)
*KfACTIN*	Kaladp0071s0282	As reference gene	TTCGAGACCTTCAATGCTCCT	GATGGCTGGAAAAGCACCTCA
*KfPHYA*	Kaladp0034s0172	Phytochrome A, flowering promoted gene [*Kalanchoe fedtschenkoi*]	TTCAGCCATTTGGGTGTTTGT	CGTATCACACAGCTTGTCCAG
*KfPHYB*	Kaladp0039s0298	Phytochrome B, flowering suppressor gene [*Kalanchoe fedtschenkoi*]	CACATGCTCTCTCTGACTCCC	AAAAGCTGAATGTCCCCTCCA
*KfCRY1*	Kaladp0071s0308	Cryptochrome 1, flowering promoted gene [*Kalanchoe fedtschenkoi*]	TCTTGGCGCCAGTTTCATCA	TGTCAGCATTGCTCCATCCA
*KfFT*	Kaladp0099s0141	PROTEIN FLOWERING LOCUS T (FT), regulation of flower development [*Kalanchoe fedtschenkoi*]	AGGGAAGTTGCAAATGCGTG	GGAAGTTCTGACGCCATCCC
*KfFPF-1*	Kaladp1115s0001	FLOWERING-PROMOTING FACTOR 1-like PROTEIN 1-related [*Kalanchoe fedtschenkoi*]	AGCGCTGATCAAAACGGTAGA	GAAAAATCGCGAGGAAGGGA
*KfPEF-4*	Kaladp0059s0037	PROTEIN EARLY FLOWERING 4 [*Kalanchoe fedtschenkoi*]	GGCGGCGGAGATATTTGTGA	TGGCAGCACGATGATGAAAG

**Table 2:** The effects of the supplementary and night-interrupting 10 μmol∙m^−2^∙s^−1^ PPFD B light on the morphological characteristics of kalanchoe (*Kalanchoe blossfeldiana* ‘Rudak’) after 60 days of exposure to the photoperiodic light treatments.

Photoperiod (I)	Blue Light (II)	Shoot							
		Plant Height (cm)	Canopy Diameter (cm)	No. of Nodes	No. of Branches	Length of Top 4th Internode (mm)	Stem Diameter (mm)	Fresh Weight (g)	Dry Weight (g)
Short day 10 h (SD)	None	13.1 ± 0.17 d ^1^	13.0 ± 0.24 e	7.4 ± 0.13 c	1.3 ± 0.04 d	14.9 ± 0.51 b	6.1 ± 0.11 d	66.6 ± 1.17 f	2.8 ± 0.05 f
	+ 4B	15.1 ± 0.20 c	14.7 ± 0.23 d	10.6 ± 0.15 b	2.9 ± 0.04 cd	13.3 ± 0.47 c	7.8 ± 0.17 c	98.3 ± 1.03 e	3.9 ± 0.05 e
	+ NI-4B	15.2 ± 0.23 c	16.6 ± 0.15 c	10.9 ± 0.15 b	3.4 ± 0.05 c	13.1 ± 0.38 c	7.6 ± 0.20 c	121.1 ± 1.98 d	5.0 ± 0.04d
Long day 13 h (LD)	None	17.7 ± 0.16 b	17.7 ± 0.20 bc	10.8 ± 0.17 b	6.6 ± 0.12 b	15.9 ± 0.27 a	8.5 ± 0.14 b	135.9 ± 2.03 c	6.0 ± 0.09 c
	+ 4B	18.6 ± 0.23 ab	18.6 ± 0.32 b	13.1 ± 0.24 a	5.8 ± 0.10 bc	14.0 ± 0.36 b	9.7 ± 0.24 a	155.2 ± 2.17 b	7.2 ± 0.09 b
	+ NI-4B	20.7 ± 0.27 a	21.5 ± 0.26 a	13.7 ± 0.21 a	8.7 ± 0.25 a	14.2 ± 0.41 b	9.9 ± 0.23 a	169.5 ± 1.74 a	8.0 ± 0.08 a
*F*-test	I	***	***	***	***	***	***	***	***
	II	***	***	***	**	**	**	***	***
	I × II	***	***	***	***	***	***	***	***
Photoperiod (I)	Blue light (II)	Leaf			Flower		Root		
		Number	Length (cm)	Width (cm)	Days to visible flower buds	No. of inflorescences	Length (cm)	Fresh weight (g)	Dry weight (g)
Short day 10 h (SD)	None	18.3 ± 1.34 e	7.1 ± 0.34 b	6.2 ± 0.20 ab	17.0 ± 1.17 c	6.7 ± 0.98 b	12.7 ± 0.57 d	4.8 ± 0.06 c	0.5 ± 0.01 c
	+ 4B	24.7 ± 2.43 d	6.4 ± 0.41 bc	5.2 ± 0.23 b	24.7 ± 1.13 b	11.3 ± 1.01 ab	15.3 ± 0.71 cd	8.5 ± 0.07 bc	0.9 ± 0.02 bc
	+ NI-4B	30.7 ± 2.17 c	6.6 ± 0.52 bc	5.5 ± 0.32 b	24.3 ± 1.03 b	12.3 ± 1.12 ab	16.0 ± 0.67 c	9.1 ± 0.05 b	1.1 ± 0.02 b
Long day 13 h (LD)	None	51.8 ± 5.44 b	8.4 ± 0.43 a	7.2 ± 0.24 a	-	0.0 ± 0.00 c	17.2 ± 0.54 b	16.1 ± 0.10 a	2.1 ± 0.02 a
	+ 4B	53.3 ± 6.27 b	7.6 ± 0.37 ab	6.4 ± 0.31 ab	33.3 ± 1.24 a	14.3 ± 1.17 a	18.4 ± 0.63 ab	15.9 ± 0.10 a	1.9 ± 0.02 a
	+ NI-4B	68.1 ± 5.72 a	7.5 ± 0.31 ab	6.5 ± 0.27 ab	-	0.0 ± 0.00 c	19.21 ± 0.91 a	16.0 ± 0.09 a	2.0 ± 0.01 a
*F*-test	I	***	***	**	***	***	***	***	***
	II	***	**	**	***	***	***	**	**
	I × II	***	**	**	**	***	***	***	***

^1^ Mean separation within columns by the Duncan’s multiple range test at *p* ≤ 0.05 and the mean ± standard error (*n* = 6). ** and ***, significant at *p* ≤ 0.01 and 0.001, respectively. Each treatment was measured six times. (See Figure 2 for details on the photoperiodic treatments with B light).

**Table 3:** The effects of the supplementary and night-interrupting 10 μmol∙m^−2^∙s^−1^ PPFD B light on the photosynthetic and chlorophyll fluorescence characteristics of kalanchoe (*Kalanchoe blossfeldiana* ‘Rudak’), after 60 days of exposure to the photoperiodic light treatments.

Photoperiod (I)	Blue Light (II)	Pn ^2^ (μmol CO_2_ m^−2^s^−1^)	Tr ^3^ (mmol H_2_O m^−2^s^−1^)	Gs ^4^ (mol H_2_O m^−2^s^−1^)	Ci ^5^ (μmol CO_2_ mol^−1^)	*F*v/*F*m ^6^	*F*v’/*F*m’ ^7^
Short day 10 h (SD)	None	13.0 ± 0.63 e ^1^	1.59 ± 0.014 cd	0.39 ± 0.013 cd	357.5 ± 10.21 cd	0.75 ± 0.017 bc	0.44 ± 0.011 bc
	+ 4B	16.3 ± 0.57 d	2.03 ± 0.011 b	0.76 ± 0.014 b	406.9 ± 10.24 b	0.80 ± 0.013 ab	0.52 ± 0.023 ab
	+ NI-4B	17.2 ± 0.87 cd	2.18 ± 0.082 a	0.81 ± 0.010 ab	487.7 ± 17.21 a	0.85 ± 0.016 a	0.58 ± 0.019 a
Long day 13 h (LD)	None	17.8 ± 0.52 c	1.76 ± 0.034 c	0.48 ± 0.013 c	380.2 ± 11.37 c	0.78 ± 0.010 b	0.48 + 0.013 b
	+ 4B	19.9 ± 0.86 b	2.08 ± 0.024 b	0.77 ± 0.011 b	413.9 ± 11.56 b	0.81 ± 0.011 ab	0.52 ± 0.024 ab
	+ NI-4B	21.9 ± 0.91 a	2.22 ± 0.083 a	0.86 ± 0.012 a	499.2 ± 15.33 a	0.86 ± 0.015 a	0.59 ± 0.021 a
*F*-test	I	***	*	*	*	**	**
	II	***	***	***	***	**	**
	I × II	***	*	**	*	NS	NS

^1^ Mean separation within columns by Duncan’s multiple range test at *p* ≤ 0.05 and the mean ± standard error (*n* = 6). ^2^ Net photosynthetic rate (*P*n). ^3^ Transpiration rate (*T*r). ^4^ Stomatal conductance (*G*s). ^5^ Intercellular CO_2_ concentration (*C*i). ^6^ The maximal PSII quantum yield (*F*v/*F*m). ^7^ The photochemical efficiency of PSII (*F*v′/*F*m′). NS, *, **, ***; non-significant or significant at *p* ≤ 0.05, 0.01, 0.001, respectively. Each treatment was measured six times. (See Figure 2 for details on the photoperiodic treatments with B light).

## Data Availability

Data is contained within the article.
